# An Internet-Based Intervention to Alleviate Stress During Social Isolation With Guided Relaxation and Meditation: Protocol for a Randomized Controlled Trial

**DOI:** 10.2196/19236

**Published:** 2020-06-17

**Authors:** Silvia Maria Francesca Pizzoli, Chiara Marzorati, Davide Mazzoni, Gabriella Pravettoni

**Affiliations:** 1 Department of Oncology and Hemato-Oncology University of Milan Milan Italy; 2 Applied Research Division for Cognitive and Psychological Science European Institute of Oncology (Istituto di Ricovero e Cura a Carattere Scientifico) Milan Italy

**Keywords:** relaxation, guided meditation, internet-based intervention, social isolation, stress, COVID-19, mental health, public health

## Abstract

**Background:**

Psychophysiological stress and decreased well-being are relevant issues during prolonged social isolation periods. Relaxation practices may represent helpful exercises to cope with anxiety and stressful sensations.

**Objective:**

The aim of this research protocol is to test whether remote relaxation practices such as natural sounds, deep respiration, and body scan meditation promote relaxation and improved emotional state and reduce psychomotor activation and the preoccupation related to the coronavirus disease (COVID-19) pandemic.

**Methods:**

The study population will consist of 3 experimental groups that will randomly receive one of 3 internet-based audio clips containing a single session of guided breathing exercise, guided body scan exercise, or natural sounds. The participants will listen to the fully automated audio clip for 7 minutes and complete pre-post self-assessment scales on their perceived relaxation, psychomotor activation, level of worry associated with COVID-19, and emotional state. At the end of the session, the participants will also be asked to provide qualitative reports on their subjective experiences.

**Results:**

Analyses will be performed to test the differences in the efficacy of the different audio clips in an internet-based intervention on 252 participants (84 per group), investigating whether natural sounds or remote guided practices such as deep respiration and body scan meditation positively enhance the participants’ perceived psychological state.

**Conclusions:**

The study will provide information on if and to what extent guided practices can help in reducing psychological side effects related to social isolation during the COVID-19 pandemic.

**International Registered Report Identifier (IRRID):**

PRR1-10.2196/19236

## Introduction

### Background

The world is facing a new health emergency: coronavirus disease (COVID-19), an infectious disease caused by a newly discovered coronavirus [1].

The COVID-19 pandemic is the first pandemic to occur in the 21st century. Therefore, the majority of the world's population is living with an entirely new experience that has never happened in their lifetime. Since mid-March, Italy has faced a strict lockdown. For an extended period of time, people have been forced to live either alone or in “bubbles” with only a few family members or a partner. In such conditions, it is not difficult to assume that people will feel lost and will experience loneliness and social isolation. These conditions may reduce health and mental well-being, thus affecting vital functions (eg, sleep quality), social connectedness, perceived support, and psychological status [2-4]. A recent rapid review on the effects of the quarantine [5] underlines that society is facing several negative cognitive and emotional problems, such as confusion, poor concentration, irritability, insomnia, distress, frustration, and anger. People are worried about the quarantine duration, insufficient information provision, economic problems, and stigma. These negative effects may impact individuals’ bio-psycho-social functioning and lead to depressive or posttraumatic stress symptoms [5,6].

The lack of a vaccine for COVID-19, the high chance of contagion, and the severity of symptoms—which often lead to death—increase risk perception related to the pandemic. In all high-risk situations, cognitive and rational thinking interact with emotional appraisals, thus affecting people’s state of mind; individuals feel vulnerable and may experience fear for themselves and for their loved ones [7,8]. Both physical and psychological dimensions are affected by the sense of uncertainty and the threat of contracting the virus. Under such conditions, it is not uncommon for people to also experience psychophysiological hyperarousal, which causes them to pay excessive attention to their bodily sensations and enhances their perceptions [9]. Overall, COVID-19 and social isolation have led to negative side effects, causing widespread concern and psychophysiological reactions [10].

Starting from these premises, it is necessary to develop an intervention to investigate people’s psychosocial conditions and reduce possible psychophysiological activation; due to the current circumstances, such an intervention must be internet-based.

Previous studies demonstrated that interventions based on natural sounds, respiration, and meditation may help individuals to alleviate the effects of stress by reducing physiological arousal and restoring autonomic balance [11-14]. In fact, listening to natural sounds significantly reduces human stress processes [13,15]. On the other hand, guided relaxation techniques are widely used to produce a deep state of relaxation and enhance physical and emotional well-being. Deep breathing exercises and focusing attention on body perception are two of the main techniques used to reduce hyperarousal and achieve a more relaxed condition. Deep breathing can be also defined as “an efficient integrative body-mind training for dealing with stress, anxiety and psychosomatic conditions” [16]; it may help people to slow their breathing, take in more oxygen, and reduce the use of their shoulder, neck, and upper chest muscles, thus achieving better emotional balance and social adaptation [17]. On the other hand, body scan meditation aims to focus attention on different parts of the body and help people become aware of their bodily sensations, such as pain, tension, warmth, or relaxation [18,19]. We chose to employ these two techniques because they involve different cognitive and psychophysiological processes. In the present study protocol, we aim to test and compare the efficacy of natural sounds, breathing regulation, and body scan meditation to assess which intervention is the most effective for the target population. Applying these interventions to people forced into mandatory social isolation may help them become more aware of their mind-body condition and reduce negative effects. Moreover, scientific studies have shown that online relaxation techniques as well as in-person programs achieve significant results; these findings may support the implementation of remote interventions, which are currently a necessary feature of proposed programs [20,21]. However, a comparison of natural sounds, respiration, and body scan meditation techniques in internet-based interventions is still lacking in the literature.

### Objective

The aim of this study is to test the difference in the efficacy of three audio clips related to three relaxation practices (deep breathing, body scan meditation, and natural sounds). We expect to find a decrease in the participants’ levels of psychomotor activation/stress and of preoccupation with thoughts related to COVID-19 as well as enhanced relaxation levels and emotional state after exposure to all the audio clips; we also expect that the guided techniques (deep breathing and body scan) will have greater effects on the abovementioned dimensions than natural sounds.

## Methods

### Participants and Procedure

The invitation to take part in the study will be published on social media webpages (WhatsApp, Facebook, LinkedIn, and Instagram). The readers will be informed about the general aim of the study and that the study is conducted by researchers from the University of Milan. Potential participants will be encouraged both to take part in the study and to share the invitation with their acquaintances. The invitation will contain a link to the Qualtrics platform, where a more detailed description will be available.

The eligibility criteria to take part in the study will include being more than 18 years of age, being a proficient Italian speaker, not suffering from any impairment affecting auditive abilities, and having an appropriate level of computer literacy. Before taking part in the study, participants will be asked to read and complete an online consent form. The document will be shown and will be downloadable in .pdf format, and the participant will be asked to give their consent online. The document has already been redacted according to national legislation and by following the guidelines of the Ethical Committee of the first author’s university.

The participation in the study will consist of 3 main parts: 1) completing a short questionnaire requesting background information and a preintervention evaluation, 2) listening to a 7-minute audio clip, and 3) completing the post-intervention evaluation. The estimated time for participating in the study (completing the three parts) will range from 12 to 17 minutes.

Participation in the study will be voluntary, and participants will be allowed to withdraw from the study at any moment. The research protocol follows the CONSORT-EHEALTH V1.6 Guidelines [22]. The study will be conducted according to the principles stated in the Declaration of Helsinki (59th WMA General Assembly, Seoul, 2008).

### Measures and Design

After completing the sociodemographic form, participants will be asked to report if they suffer from a chronic disease and how much the disease impacts their perceived vulnerability to COVID-19. Participants will also be asked to report their working situation, recent changes in occupational status due to COVID-19 restrictions, and if they have prior experience with relaxation techniques. Then, participants will be asked to complete 3 self-assessment questionnaires aimed at measuring their current level of anxiety, their tendency to worry about physical signals and sensations, and the degree of attention they pay to their bodily feelings.

To assess the abovementioned aspects, the following questionnaires will be used: the State-Trait Anxiety Inventory form-trait subscale (STAI-Y) (20 items on a 4-point Likert scale from 1=not at all to 4=very much) [23,24]), which assesses trait anxiety; the subscale physical concerns of the Anxiety Sensitivity Index-3 (ASI-3) [25,26], composed of 6 items that require them to rate how much they worry about physical sensations on a 5-point Likert scale from 1=very little to 4=very much; and finally, the Body Vigilance Scale (BVS) [27], a 4-item scale in which they will describe how much they usually pay attention to body sensations on a scale from 0=”not at all like me” to 10=“completely like me”. In the fourth item, participants will be required to rate their attention to 15 body sensations that are the core physical symptoms of panic attacks [28].

After completing the questionnaires, participants will be randomly assigned to one of the three experimental groups via the randomization procedure within Qualtrics. Specifically, in each experimental condition, participants will receive a 7-minute recorded audio clip aimed to promote a state of awareness and relaxation. In the first experimental condition (ie, square breathing), participants will hear a recorded voice that guides the regulation of breathing frequencies with the aim of making every breathing act (inhalation, holding breath, exhalation, and holding breath) last the same amount of time (4 seconds). In the second experimental condition (ie, body scan meditation), an audio clip with a voice that guides the participant’s attention through every part of the body will be presented, and the listener will be invited to feel tensions and unpleasant feelings and to let them go. Both tracks were recorded by a trained mindfulness and yoga expert in collaboration with a psychotherapist and were pretested on 4 participants to assess the ease and the perceived effectiveness of the exercise. In the third experimental condition (ie, natural sounds), participants will be stimulated by a prerecorded audio clip of natural sounds. All the audio clips will be preceded by instructions on the recommended place and body position for the exercises.

As pre-post measures, before and after the audio stimuli, the participants will be asked to self-rate their perceived relaxation level, perceived stress level, or psychomotor activation degree, to rate how concerned they feel about COVID-19, and to rate 3 specific features of their emotional state. Specifically, participants will be also asked to rate on a 3-item Visual Analogue Scale (VAS) (0=not at all to 10=completely) how relaxed they feel, how stressed/activated they feel, and how much their thoughts related to COVID-19 scare them; furthermore, they will complete the Self-Assessment Manikin (SAM) [29] for emotional states, which is a 3-item visual scale (valence, intensity/arousal, and dominance) commonly used to quantify properties of a person’s overall emotional state on a scale of 1 to 5 images. Immediately after listening to the audio, participants will also be asked if they heard the entire audio track, a part of it, or no part.

Finally, all the participants will have the opportunity to describe their personal experience and to provide suggestions for future changes. Specifically, participants will be asked to write a short paragraph answering 2 open-ended questions about their personal experience with the exercise (“Did you enjoy or dislike the experience with the audio clip?”) and any changes they would appreciate (“Would you change something in the audio clip?”).

### Data Analysis

Group differences in demographic data and pretreatment measures will be analyzed with one-way analysis of variance (ANOVA) followed by *t* tests with Bonferroni corrected *P* values and chi-square tests. Differences in questionnaire scores (ie, STAI-Y, ASI-3, BVS) between groups will be examined using the same analysis. STAI scores will also be compared to clinical cutoffs. The internal consistency of the scales (Cronbach α) in our sample will also be assessed.

As the main aim of this study is to test the difference in efficacy between audio clips, our main analysis will be one-way ANOVA on gain relaxation scores [30], with 3 groups and fixed effects and with no interaction. According to our main objective, we performed a priori sample size calculation, considering a 0.25 effect size on the level of relaxation, with a high statistical power (0.95) and an alpha of .05. Considering the specified values, we will need a total sample size of 252 participants with complete surveys to reach the desired statistical power (84 per group). In view of the possibility of attrition of participants, which may occur in the present electronic health (eHealth) trial in the form of incomplete questionnaires or dropout before listening to the complete audio clips, we estimate that we will obtain incomplete data for 30% of the participants who will start the survey by clicking on the link and who will not continue with the questionnaires; thus, we should register a total of 328 participants.

Independent sample *t* tests of the relaxation level between paired groups will also be carried out on participants who will complete the entire survey (eg, without considering participants who dropped out before the randomized exposure to the audio clips).

The same analysis will be conducted for the participants’ perceived stress and fear related to COVID-19 thoughts, assessed with the VAS continuous scale, and for their emotional states, measured through the SAM. The between-group post-exercise Hedges g effect size (CI 95%) will be computed. As the randomization will be automatically performed by Qualtrics, the researchers who analyze the data will be blinded to which audio clip participants received.

Furthermore, we will perform other explorative analyses to test if and to what extent other covariates impact the efficacy of the proposed techniques. Specifically, we will test if having job issues or a chronic disease has an impact on the efficacy of the techniques.

All the aforementioned analyses will be performed in SPSS version 26.0 (SPSS, Inc., Chicago, IL).

Lastly, qualitative reports on subjective experiences and suggestions will be analyzed to understand the participants’ subjective experiences, to evaluate the perceived efficacy of the audio, and to identify possible ameliorations of and issues with the proposed stimuli. Practical advice for future studies will be given accordingly.

## Results

The expected results are that the audio clips will be effective in reducing the participants’ perceived stress and degree of preoccupation with thoughts related to COVID-19; we also believe that the guided techniques will have a greater effect on relaxation and emotional state enhancement compared to the natural sounds.

The research protocol was approved by the Ethical Committee of the first author’s university on April 30, 2020. Recruitment started in May 2020.

## Discussion

People are confronting the COVID-19 pandemic worldwide. This alarming event and the unknown consequences it will trigger in citizens’ lives have led to increases in stress levels and psychophysiological arousal. Moreover, lockdowns have forced people into social isolation and are preventing the implementation of in-person programs to target psychological issues.

The present randomized study will provide data on the effectiveness of remotely delivered interventions with natural sounds, deep breathing, and meditation practice in improving people’s perceived relaxation, psychomotor activation, level of worries associated with COVID-19, and emotional state; we will also obtain data on people’s experiences with these techniques.

The study will also shed light on whether one of these exercises provides increased benefits compared to the others and if so, to what extent. Indeed, a comparison among natural sounds, respiration, and body scan meditation techniques in internet-based interventions is lacking in the literature.

As a first assessment of the efficacy of these approaches, the present study will have the possible limitation of being a single-session guided intervention. Thus, we will not be able to assess differences in the efficacy of more prolonged exposure to the relaxation sessions. Furthermore, the length of the audio clips may need to be adjusted in future interventions in light of the participants’ reports.

If effective, this study could guide the development of future low-cost remote interventions to reduce worries and anxiety in the general population. Future studies may also assess and compare the efficacy of these approaches in clinical protocols for patients struggling with anxiety and hyperarousal.

## Abbreviations

ASI-3: Anxiety Sensitivity Index-3
BVS: Body Vigilance Scale
COVID-19: coronavirus disease
eHealth: electronic health
SAM: Self-Assessment Manikin
STAI-Y: State-Trait Anxiety Inventory form-trait subscale
VAS: Visual Analogue Scale
